# Determinants of Patients' Choice of Provider in Accessing Brucellosis Care among Pastoral Communities Adjacent to Lake Mburo National Park in Kiruhura District, Uganda

**DOI:** 10.1371/journal.pone.0105276

**Published:** 2014-08-18

**Authors:** Catherine Kansiime, Elizeus Rutebemberwa, Anthony Mugisha, Samuel Mugisha, Benon B. Asiimwe, Innocent B. Rwego, Suzanne N. Kiwanuka

**Affiliations:** 1 Department of Health Policy Planning and Management, Makerere University School of Public Health, College of Health Sciences, Kampala, Uganda; 2 School of Veterinary Medicine and Animal Resources, Makerere University, Kampala, Uganda; 3 Department of Biological Sciences, College of Natural Sciences, Makerere University, Kampala, Uganda; 4 Department of Medical Microbiology, College of Health Sciences, Makerere University, Kampala, Uganda; 5 Ecosystem Health Initiative, College of Veterinary Medicine, University of Minnesota, St. Paul, Minnesota, United States of America; National University, Costa Rica

## Abstract

**Background:**

Brucellosis is the commonest zoonotic infection worldwide with symptoms similar to other febrile syndromes such as malaria and typhoid fever. It is often easily misdiagnosed, resulting in underreporting and misdirected treatments. Understanding of the factors that influence brucellosis care seeking is essential in enhancing its effective management. Our study sought to determine the factors associated with choice of provider in accessing care for brucellosis among pastoral communities in Uganda.

**Methods:**

This was a cross-sectional survey involving 245 randomly selected respondents previously diagnosed and treated for brucellosis, two months before the study. They were enrolled from three sub-counties neighboring Lake Mburo National Park between December 2012 to April 2013. Data on socio-demographics, availability, accessibility, affordability and acceptability of health services were collected. A multivariable logistic regression model was fitted to determine association between independent and outcome variables using odds ratios and 95% confidence intervals with p-value≤0.05 considered statistically significant.

**Results:**

Of the 245 respondents, 127(51.8%) sought health care at government facilities and the rest at private. Respondents who were less likely to choose a government facility were either single (OR: 0.50, CI: 0.26–0.97), had general weakness (OR: 0.09, CI: 0.01–0.72) or whom family took a decision (OR: 0.52, CI: 0.28–0.97). At multivariable analysis, choice of government facility was influenced by primary education (aOR: 0.46, CI: 0.22–0.97), having six to ten household members (aOR:3.71, CI:1.84–7.49), family advice (aOR:0.64, CI: 0.23–0.91), distance ≥10 kms (aOR:0.44, CI: 0.21–0.92), high costs at private clinics (aOR:0.01, CI:0.02–0.15) and no diagnosis at government facility (aOR:0.11, CI:0.01–0.97). Females were more likely to seek health care at government facilities, while those with tertiary education were less likely, after the first provider.

**Conclusions:**

Females and households with six to ten members were more likely to choose government facilities. Government facilities need to be equipped to attract more patients.

## Introduction

Brucellosis is among the most widespread zoonotic infections causing human suffering and economic losses in livestock [1]. The disease is considered the commonest zoonotic infection worldwide [2] with more than 500,000 cases recorded annually [3]. However, it is often a neglected cause of morbidity in many regions of the world [4]. In sub-saharan Africa, prevalence of 5-55% in humans and 8–46% in animals reported [5] and in Uganda, human brucellosis has been reported to be prevalent in both rural and urban areas [6]. In sub-Saharan Africa, brucellosis is often easily misdiagnosed as other febrile syndromes such as malaria and typhoid fever, thereby resulting in underreporting and hence misdirected treatments [7]. Access to health care thus comes into play to address the health needs of the people. Whereas, there is no universally accepted definition of access to health services [8], in this study we used the definition of ‘the timely use of services according to need [9]. However, various barriers to accessing health services have been highlighted, and they stem from the demand side and/or the supply side [10], [11].

Determining choice of provider is often an interplay of numerous factors [12]. Some of the factors include; availability, affordability, physical accessibility or acceptability and adequacy of services [13]. Family size and parity, educational status and occupation of the head of the family, age, gender and marital status [14]. Other barriers are perceived lack of skilled staff in public facilities, late referrals or non-referrals to more specialized care, health worker attitude, costs of care and lack of knowledge about the disease by patients and health workers [15]. Access to and utilization of health services in Uganda has improved over both Health Sector Strategic Plan periods [16], with improvements in physcial access to health facilities from 49% (2001) to 72% (2004) of the population living within 5 km of a health facility. In Uganda, previous studies on access to care have focused on febrile illnesses [18] and fever [19], while similar studies focused on utilization of health services by the poor [20], [21]. To date, no study has been done in Uganda to determine access to care and factors that influence choice of provider for brucellosis, yet understanding of these factors is essential in order to enhance effective management of the disease. This study therefore aimed at determining factors associated with choice of provider in accessing care for patients diagnosed of brucellosis among pastoral communities adjacent to Lake Mburo National Park (LMNP) in Uganda.

## Methods

### Study area and population

The study was conducted in the pastoralist rangelands (natural landscapes in the form of grasslands, shrub lands, and woodlands) of Nyabushozi county of Kiruhura district in three purposively selected sub-counties of Kanyaryeru, Nyakashashara and Sanga which are adjacent to Lake Mburo National Park. The study area has one government Health Centre (HC) IV, and in each sub-county, there is one Health Center I at the village level which acts as an outpost for outreach services, one Health Centre II and one Health Centre III which mainly provide outpatient primary health care. However, none of the Health Centre IIIs has a functional laboratory to test for brucellosis but all the government facilities have clinical officers or nurses for health care delivery. There are also a number of other providers such as private health facilities which include private for profit clinics, drug shops, dispensaries and a few traditional healers. The majority of the people in this area are agro-pastoralists and farmers with a few semi-nomads.

### Study design and sampling procedure

A cross-sectional study was conducted between December 2012 to April 2013 and 245 households with a member who had been diagnosed and treated for brucellosis two months prior to the study and these were enrolled after consenting. A semi-structured administerd questionnaire was used to obtain information on access to health care such as barriers and facilitating factors that influenced their choice of health provider as well as socio-demographic factors. The sample size was based on the formula for Cross-sectional studies [Z^2^P(1-*P*)/D^2^] [22]. *P* is prevalence and *d* is the level of significance (0.05). Expected prevalence of choice of provider of brucellosis patients who gave a history of going to a hospital as the first point of contact was 87.7% [23]. This yielded a sample size of approximately 169 respondents which was adjusted for, a 15% non-response rate with a design effect of 1.5 was calculated and yielded an estimated sample size of 291 households. Ninty seven households were proportionately sampled from each of the three sub-counties.

### Conceptual framework

To examine and interprete the findings in our study, we used the Health Access Livelihood Framework described by Obrist et, 2007 [23] that provides an outline within which we can consider health service approach and health seeking approaches in relation to the five dimensions (5As) of access that influence the course of the health seeking process. These are; availability, accessibility, affordability, adequacy and acceptability of services and examines why, when and how individuals and communities seek access to health care services in light of potential livelihood assets and actions such as financial, social, human, natural and physical capital [23].

### Data Collection and Management Procedures

Structured interviewer administered questionnaires were used to collect data on the five dimensions that influence access to health care: availability, accessibility, affordability, adequacy, and acceptability and these included questions on; distance to the health facility, availability of brucellosis drugs, health workers' attitude, waiting hours, second choice of provider and costs of care. Individual socio-demographic factors such as age, sex, religion, occupation, education, household size and marital status were also collected. Other factors were reasons for choosing a provider, severity of disease, who decided where to seek care and family support. The dependent variable was choice of provider (government facility and private clinics). Three Research Assistants were trained, all the study instruments were pre-tested before the study begun, the questionnaire was translated and back translated from the original English version into the local language (Runyankole) to ensure consistency and validity. All the questionnaires were checked for completeness during data collection. Collected data was entered into Epi-data version 3.1 to check for errors and to ensure accuracy and then exported to STATA statistical soft ware version 11 for analysis.

### Data analysis

At univariate analysis categorical variables were summarized into frequencies and percentages. Chi square tests were used for categorical variables and fisher's exact test was used for variables that had cells less than 5. At bivariate analysis, categorical variables were assessed for the association between the independent variables and choice of provider (categorized into government and private health facility) using Odds Ratios (ORs) and 95% Confidence Intervals (CIs). Variables *p*≤0.2 were taken for multivariable analysis. We used cut-off of ≤0.2 because we did not want to include any unnecessary variables but at the sametime, we did not want to miss out important variables, however, this is not a static value. A multivariable logistic regression model analysis was fitted to determine the association between independent and outcome variables. Inclusion of variables into the multivariable analysis was based on factors in bivariate analyses that had *p*≤0.2 or *p*≤0.05 or variables that are known to be potential confounder from previous studies.We found no confounders. A *p*-value≤0.05 was considered statistically significant. All the above steps were done during sub-analysis for those who sought care after the first provider and the outcome of interest at this stage was choice of second provider (categorized into government and private health facility). Model validity was assessed using the Hosmer-Lemeshow goodness-of-fit test with a *p*<0.71, which means it was a good model.

## Ethics Statement

The study protocol was approval by Makerere University School of Public Health Higher Degrees, Research and Ethics Committee as well as Uganda National Council for Science and Technology. The study objective was explained to participants in their local language (Runyankole) and informed written consent was obtained from each study participant who agreed to participate in the study. Each participant was interviewed independently and the collected information was coded for anonymity and confidentiality was assured during interviewing.

## Results

### Socio- demographics and other factors

A total of 245 respondents from three sub-counties (Kanyaryeru, Sanga and Nyakashashara) in Kiruhura district were recruited into the study. Of these, 127(51.8%) sought health care at a government facility while 118 (48.2%) at a private health facility. Majority were between the age of 18 to 41, females were 161(65.7%). About 49.4% had attained primary education. Most of the respondents were agro-pastoralists and farmers. The main source of income was cattle keeping 81(33.1%) and farming 73(29.8) and one hundred and thirty-two (53.9%) of the respondents had six to ten members in their households (Table 1).

**Table 1:** Socio-demographic factors of respondents.

Variables	Frequencies (245)	Percentages (%)
**Sex**		
Male	84	34.3
Female	161	65.7
**Age**		
18–29	86	35.1
30–41	81	33.1
42–53	40	16.3
54–65	26	10.6
>66	12	4.9
**Religion**		
Christian	221	90.2
Moslem	24	9.8
**Marital status**		
Married	168	68.6
Single	48	19.6
Divorced/separated	11	4.5
Widowed (er)	18	7.3
**Education**		
Informal	68	27.8
Primary	121	49.4
Secondary	46	18.8
Tertiary	10	4.08
**Occupation**		
Farmer	72	29.4
Agro-pastoralist	81	33.1
Trader/business	40	16.3
Others**	52	21.2
**Main source of income**		
Salary	12	4.9
Trading	37	15.1
Farming	73	29.8
Cattle keeping	81	33.1
Others**	42	17.1
**Number of household members**		
1–5	87	35.5
6–10	132	53.9
>10	26	10.6

**Other occupation included: student, teacher, unemployed, security guard and boda boda cyclist. Other source of income include: none, mechanic, bodaboda and butcher man.

The three main reasons given that influence choice of a government provider were first of all; better services 44(34.7%) which included availability of diagnostic tests and treatment of human brucellosis. The second was nearest health facility 36(28.3%), and thirdly if they thought it was general weakness 3(27.3%). The problems that that hinder accessing care from the government facilillty were; long waiting hours 46(36.2%) and unavailability of diagnostic tests and treatment 44(34.6%). High costs of diagnosis and treatment 47(39.8%) was a barrier in accessing care at the private facility. More than half of the respondents (60.2%) sought health care at a private facility when the disease was severe. Majority of the respondents 181(73.9%) mentioned obtaining family support in seeking for health care at both government 94(74%) and private facilities 87(73.7%). More than a half of the respondents who sought care at the private facility 70(59.3%) and government facility 68(54%) mentioned unsupportive health providers as a barrier in accessing care.

### Association of participants' socio-demographic characteristics and other health factors with choice of provider

During bivariate analysis, all variables that were less or equal to *p* values of 0.05 and 0.2 as shown in tables 2 and 3 were taken for multivariable analysis. Variables with *p* value≤0.2 included; socio-demogrphic factors like sex and occupation; availability and accessibility factors like severity of disease, family support, and distance to health facility; Affordability factors such as how money was raised, and time taken to reach health facility; Acceptability factor such as conduct of health providers.

**Table 2:** Association of participants' socio-demographic characteristics with choice of provider.

	Choice of provider, n (%)			
Variables	Private health facility 118(48.2%)	Government health facility 127 (51.8%)	Unadjusted OR(95%CI)	*P*-value
**Age**				
18–29	46(39)	40(31.5)	1	
30–41	37(31.4)	44(34.7)	1.37(0.74–2.51)	0.31
42-53	15(12.7)	25(19.7)	1.92(0.89–4.13)	0.09
54–65	14(11.9)	12(9.5)	0.98(0.41–2.37)	0.97
>66	6(5.1)	6(4.7)	1.15(0.34–3.85)	0.82
**Marital status**				
Married	73(61.9)	95(74.8)	1	
Single	29(24.6)	19(15)	0.50(0.26–0.97)	0.04*
Divorced/separated	6(5.1)	5(3.9)	0.64(0.19–2.18)	0.48
Widowed	10(8.5)	8(6.3)	0.61(0.23–1.64)	0.33
**Education attained**				
No formal education	25(21.2)	43(33.9)	1	
Primary	66(55.9)	55(43.3)	0.48(0.26–0.89)	0.02*
Secondary	21(17.8)	25(19.7)	0.69(0.32–1.48)	0.34
Tertiary	6(5.1)	4(3.1)	0.39(0.09–1.51)	0.71
**Number of household members**				
1–5	52(44.1)	35(27.6)	1	
6–10	54(45.8)	78(61.4)	2.15(1.24–3.72)	0.01*
>11	12(10.1)	14(11.0)	1.73(0.72–4.19	0.22
**Symptom (Fatigue)**				
No	113(96.6)	113(89.0)	1	
Yes	5(3.4)	14(11.0)	3.53(1.12–11.05)	0.03*

*Statistical significance ≤0.05.

**Table 3:** Accessibilty factors associated with choice of provider.

Variables	Choice of provider, n (%)			
Availability and accessibility factors	Private health facility 118 (48.2%)	Government health facility 127 (51.8%)	Unadjusted OR(95%CI)	*P*-value
**Reasons for choosing provider**				
Cheaper	2(1.7)	8(6.3)	1	
Advised by others	10(8.5)	18(14.2)	0.45(0.08–2.54)	0.37
Better services	38(32.2)	44(34.7)	0.29(0.06–1.45)	0.31
Did not get better	5(4.2)	5(3.9)	0.25(0.06–1.45)	0.13
Nearest facility	43(36.4)	36(28.3)	0.21(0.04–1.05)	0.06
Severity of disease	8(6.8)	7(5.5)	0.22(0.03–1.39)	0.11
Thought it was general weakness	8(6.8)	3(27.3)	0.09(0.01–0.72)	0.02*
Trust the health facility	5(3.4)	5(4.7)	0.37(0.05–2.77)	0.37
**Who decided where to seek care?**				
Myself	49(41.5)	62(48.8)	1	
Spouse	25(21.2)	33(26.0)	1.04(0.55–1.98)	0.89
Family	38(32.2)	25(19.7)	0.52(0.28–0.97)	0.04*
Others**	6(5.1)	7(5.5)	0.92(0.29–2.92)	0.89
**What family support?**	N = 88	N = 96		
Provided transport	20(22.7)	32(33.3)	1	
Money for laboratory	55(62.5)	43(44.8)	0.49(0.25–0.97)	0.04*
Provided food at the health facility	3(3.4)	8(8.3)	1.67(0.39–7.03)	0.49
Money for treatment	10(11.4)	13(13.5)	0.81(0.30–2.19)	0.68
**Affordability factors**				
**Problems encountered**				
No problem	1(0.85)	16(12.6)	1	
High costs	47(39.8)	10(7.9)	0.01(0.01–0.11)	<0.001*
Long distance	9(7.6)	11(8.7)	0.08(0.01–0.69)	0.02*
No diagnosis and treatment	36(30.5)	44(34.6)	0.08(0.01–0.60)	0.02*

*Statistical significance ≤0.05.

**others includes friends.

At bivariate, respondents who were: single (OR: 0.50, CI: 0.26–0.97), and had attained primary education OR: 0.48, CI: 0.26–0.89) were less likely to seek care at a government health facility whereas households with six to ten household members (OR: 2.15, CI: 1.24–3.72) were more likely to choose a government provider (Table 2).

Individual and social network factors at bivariate analysis included; respondents who thought it was general weakness (OR: 0.09, CI: 0.01–0.72), whom family decided where to seek care (OR: 0.52, CI: 0.28–0.97), and those that family supported in terms of providing money for diagnostic tests (OR: 0.49, CI: 0.25–0.97), these were less likely to choose a government provider (Table 3).

High costs in diagnosis and treatment (OR: 0.12, CI: 0.05–0.27) was mentioned as one of the barriers in accessing health care from a private provider. Among the brucellosis symptoms, respondents feeling fatigued (OR: 3.52, CI: 1.12–11.05) were more likely to seek care at a government facility compared to a private facility (Table 3).

### Multivariable analysis

A multivariable analysis was run for variables that were significantly associated with the choice of provider at bivariate level and those that had a *P* value≤0.2. The model was adjusted for confounding and we found no confounding. The final model after adjusting for confounding showed the following significant varaibles associated with the choice of government health provider. Households with six to ten household members (aOR:3.71, CI:1.84–7.49) were more likely to choose a government health provider whereas, respondents who had attained primary education (aOR: 0.46, CI: 0.22–0.97), whom the family decided where to seek care (aOR:0.64, CI: 0.23–0.91), distance equal or more than 10 kms (aOR:0.44, CI: 0.21–0.92), those who mentioned high costs in diagnosis and treatment of brucellosis at the private facility (aOR:0.01, CI:0.02–0.15) and no diagnosis and treatment at government (aOR:0.11, CI:0.01–0.97) as barriers to health care were less likely to choose a government health facility (Table 4).

**Table 4:** Factors associated with choice of provider at multivariable analysis.

	Choice of provider, n(%)				
Variables	Private health facility 118 (48.2%)	Government health facility 127 (51.8%)	Unadjusted OR(95%CI)	Adjusted OR(95%CI)	*P*-value
**Education attained**					
No formal education	25(21.2)	43(33.9)	1	1	
Primary	66(55.9)	55(43.3)	0.48(0.26–0.89)	0.46(0.22–0.97)	0.04*
Secondary	21(17.8)	25(19.7)	0.69(0.32–1.48)	0.65(0.25–1.70)	0.38
Tertiary	6(5.1)	4(3.1)	0.39(0.09–1.51)	0.29(0.05–1.83)	0.19
**Occupation**					
Farmer	33(28)	39(30.7)	1	1	
Agro-pastoralist	37(31.4)	44(34.6)	1.00(0.53–1.90)	1.38(0.63–3.03)	0.42
Trader/business	17(14.4)	23(18.1)	1.14(0.52–2.50)	2.21(0.87–5.60)	0.09
Others	31(26.3)	21(16.5)	0.57(0.28–1.18)	1.33(0.49–3.60)	0.57
**Number of household members**					
1–5	52(44.1)	35(27.6)	1	1	
6–10	54(45.8)	78(61.4)	2.15(1.24–3.72)	3.71(1.84–7.49)	<0.001*
>11	12(10.1)	14(11.0)	1.73(0.72–4.19)	1.60(0.52–4.95)	0.41
**Fatigue**				1	1
**No**	113(96.6)	113(90)			
Yes	5(3.4)	14(11.0)	3.53(1.12–11.05)	3.66(0.96–13.9)	0.06
**Who decided where to go?**					
Myself	49(41.5)	62(48.8)	1	1	
Spouse	25(21.2)	33(26.0)	1.04(0.55–1.98)	1.49(0.70–3.19)	0.30
Family	38(32.2)	25(19.7)	0.52(0.28–0.97)	0.64(0.23–0.91)	0.03*
Others	6(5.1)	7(5.5)	0.92(0.29–2.92)	0.77(0.13–4.54)	0.77
**Distance to facility**					
less or equal to 5 km	42(35.6)	56(44.1)	1	1	
5 to 10 km	21(17.8)	26(20.5)	0.93(0.46–1.87)	0.56(0.23–1.34)	0.19
≥10 km	55(46.6)	45(35.4)	0.61(0.35–1.107	0.44(0.21–0.92)	0.03*
**Problems encountered**					
No problem	1(0.85)	16(12.6)	1	1	
High costs at private clinic	47(39.8)	10(7.9)	0.01(0.01–0.11)	0.01(0.02–0.15)	<0.001*
Long distance	9(7.6)	11(8.7)	0.08(0.01–0.69)	0.12(0.01–1.35)	0.09
No diagnosis and treatment	36(30.5)	44(34.6)	0.08(0.01–0.60)	0.11(0.01–0.97)	0.05*
Long waiting hour	25(21.2)	46(36.2)	0.12(0.01–0.92)	0.17(0.02–1.52)	0.11

*Statistical significance ≤0.05.

### Sub- analysis for those who sought care after the first provider

A total of 126 out of 245 respondents sought care after the first provider. Of these, 91 (72.2%) and 35 (27.8%) went to government and private health facilities respectively. The total number of females was 82 (65.1%) and of these, 64 (70.3%) and 18 (51.4%) sought care from the government and private facilities respectively. Analysis was done both at bivariate and multivariable using logistic regression as mentioned in the methods section above. We found that females (aOR:5.97, CI: 1.35–26.3) were more likely to seek health care at a government facility after the first provider. Respondents who attained tertiary education (aOR:0.08, CI:0.01–0.43) were less likely to seek health care at a government health facility after the first provider compared to those who attained secondary education (Table 5).

**Table 5:** Factors associated with choosing the second health provider (Multivariable analysis).

	Choice of provider, n (%)				
Variables	Private health facility 35 (27.8%)	Government health facility 91 (72.2%)	Unadjusted OR(95%CI)	Adjusted OR(95%CI)	*P*-value
**Sex**					
Male	17(48.6)	27(29.7)	1	1	
Female	18(51.4)	64(70.3)	2.24(1.00–4.99)	5.97(1.35–26.3)	0.02*
**Education attained**					
No formal education	7(20.0)	19(20.9)	1	1	
Primary	18(51.4)	49(53.8)	1.00(0.36–2.78)	0.48(0.06–3.80)	0.49
Secondary	7(20.0)	19(20.9)	1.63(0.29–3.41)	0.07(0.01–1.42)	0.08
Tertiary	3(8.6)	4(4.4)	0.49(0.09–2.77)	0.08(0.01–0.43)	0.02*
**Occupation**					
Farmer	7(20.5)	26(28.6)	1	1	
Agro-pastoralist	18(51.4)	26(28.5)	0.39(0.14–1.09)	0.25(0.03–2.28)	0.22
Trader/business	4(11.4)	19(20.9)	1.28(0.33–4.99)	0.80(0.04–14.80)	0.88
Others	6(17.1)	20(22.0)	0.90(0.26–3.09)	0.74(0.07–8.28)	0.81
**Fatigue**					
No	29(82.9)	86(95.6)	1	1	
Yes	6(17.1)	5(4.4)	0.22(0.06–0.84)	0.01(0.01–0.29)	0.01*

*Statistical significance ≤0.05.

Multivaraible analysis using poisson regression on page 23 was conducted using logistic regression model expect that using poisson regression narrowed the 95% confidence intervals (Table 6).

**Table 6:** Multivariable analysis usin poisson regression.

	Choice of provider, n(%)				
Variables	Private health facility 118 (48.2%)	Government health facility 127 (51.8%)	Unadjusted Coef. (95%CI)	Adjusted Coef. (95%CI)	*P*-value
**Occupation**					
Farmer	33(28)	39(30.7)	1	1	
Agro-pastoralist	37(31.4)	44(34.6)	0.01(−0.29–0.29)	1.06(−0.18–0.39)	0.46
Trader/business	17(14.4)	23(18.1)	0.06(−0.28–0.40)	3.21(−0.01–0.66)	0.06
Others	31(26.3)	21(16.5)	−0.29(−0.69–0.10)	0.12(−0.28–0.51)	0.56
**Number of household members**					
1–5	52(44.1)	35(27.6)	1	1	
6–10	54(45.8)	78(61.4)	0,38(0.09–0.68)	0.49(0.20–0.77)	<0.001*
>11	12(10.1)	14(11.0)	0.29(−0.14–0.73)	0.24(−0.18–0.67)	0.41
**Fatigue**				1	1
**No**	113(96.6)	113(90)			
Yes	5(3.4)	14(11.0)	0.45(0.17–0.73)	0.34(0.03–0.66)	0.03
**Distance to facility**					
less or equal to 5 km	42(35.6)	56(44.1)	1	1	
5 to 10 km	21(17.8)	26(20.5)	−0.03(−0.34– −0.28)	−0.26(−0.55–0.04)	0.19
≥10 km	55(46.6)	45(35.4)	−0.24(−0.52– −0.04)	−0.34(−0.63– −0.05)	0.02*
**Problems encountered**					
No problem	1(0.85)	16(12.6)	1	1	
High costs at private clinic	47(39.8)	10(7.9)	−1.31(−1.89– −0.72)	−1.27(−1.83– −0.71)	<0.001*
Long distance	9(7.6)	11(8.7)	−1.16(−0.60–0.27)	−0.08(−0.48–0.32)	0.70
No diagnosis and treatment	36(30.5)	44(34.6)	−1.16(−0.43–0.10)	−0.14(−0.42–0.13)	0.30
Long waiting hour	25(21.2)	46(36.2)	0.37(0.16–0.58)	0.23(−0.13–0.60)	0.21

*Statistical significance ≤0.05. Coeff = coefficient.

## Discussion

### Availability and accessibility factors

In our study, during multivariable analysis, respondents who mentioned distance of more than 10 kms were less likely to access health care from a government health facility compared to a private one. This could be as a result of other health providers being nearer to them such as private clinics, laboratories, pharmacies and drug shops than government facilities. Studies elsewhere have found distance to the health facility to influence access to health care [19], [24] and that patient prefer providers who are near [25], [26]. A systematic review of access and utilization of health services showed that availability of drugs, distance to health facilities are the key determinants influencing health service utilization [15]. Another study in Uganda [19] found that distance to the health provider was one of the key drivers of choice of service provider. In contrast, a study in Uganda [20] found that distance was not a significant factor at multivariable analysis as a predictor for actual reported utilization. On average, although physical access to health facilities in Uganda has increased from 49% (2001) to 72% (2004) of the population living within 5 km of a health facility [17], it is clear that distance to facilities is still an issue in some communities for brucellosis patients in particular. Therefore, there is need to ensure these community members have easy access to health facilities where diagnostic and treatment services are readily available. Moreso, improved geographical access needs to be matched with good quality basic services, adequate medicines, qualified health personnel and regular supportive supervision [15] in order to improve the health status of the population.

Our study found that respondents were less likely to choose a government provider if the extended family decided where to seek health care and also provided financial support in terms of diagnostic tests. This highlights the importance of social network in seeking health care especially in terms of the African context where illness is often regarded as a social phenomenon [21]. This enables accessibility to health care services because everyone is involved in active participation in community life and mobilization of resources. A study in West Africa [27] showed that availability of social network enabled many poor people in Ivory Coast to access expensive modern health care services. Other studies highlighted social capital in terms of social network as one of the livelihood assets that influence access to health care [13]. In addition, a review of literature from Tanzania [24] found that family members and relatives provide financial, practical and moral support in seeking care especially for children. Rutherford *et al* [28] also highlighted that lack of social support was an important factor hampering access to care. With this increased importance attributed to social network in accessing health care, there is need for its promotion in communities through encouraging and promoting social capital in order to improve access to health care.

### Affordability factors

At multivariable analysis, respondents who mentioned high costs involved in the treatment of brucellosis were less likely to choose a government health facility compared to a private health facility. This may be attributed to indirect costs incurred by the service user during seeking of care such as transport, patient food, care taker accommodation and the opportunity costs derived from income foregone by the patient or care taker due to care seeking [29]. Ensor and Cooper [10] considered waiting time and direct payment for services as mixed supply-side and demand-side barriers in accessing health care. In a study done in Kenya by Kangwana *et al* [30], showed that low levels of artermisinin –based combination therapy (ACT) uptake was attributed to various factors including; high cost and frequent stock outs in the public health facilities. However, studies done in Uganda [31], [32] have found costs to be a frequent barrier in accessing services especially for the poor. In Tanzania [24], major obstacles related to affordability were; complaints about fees such as paying for drugs and ambulance transport. Poor people had to resort to short-term coping strategies like selling critical assets such as crops in order to pay for health care. Although in Uganda brucellosis treatment is free in government health facilities it is critically limited by frequent stock outs and unavailability of diagnostic equipment and reagents. Therefore, there is need to increase the demand for these better services by highlighting prompt treatment for brucellosis as well as the provision of equipped health facilities to address the demand.

### Adequacy and acceptability

At multivariable analysis, respondents who had attained primary education were less likely to choose a government health facility compared to those who had no formal education. This may be as a result of low investment in health services by the government of Uganda which falls below the estimated minimum to provide the basic health care package [16]. This has resulted in gaps in service delivery such as lack of fully functional laboratories, stock out of medicines and supplies, inadequate skilled, under-supervised health workers [20] thus resulting in the use of private rather than government facilities despite the free care in government facilities. It is not enough for government to build and bring closer more health facilities without improving and providing the essential health services in these centers. Therefore. There is need for the government to adequately support government health facilities by providing functional laboratories that are well equipped, availing regular medicines and supplies, training and motivating health workers in order to retain them in areas where they are most needed.

Respondents who had six to ten household members were likely to seek health care from a government health facility compared to a private facility. This could be because of the high costs involved in seeking care [31], [32] which influence households with many members to seek care at government health facilities that offer cheap or free treatment. Family or household size was also found to be associated with health seeking behavior [14]. Despite the free or cheap services at the government health facilities, respondents in our study were less likely to choose a government health facility because of lack of diagnostic equipment and brucellosis treatment yet the availability of essential drugs is a prerequisite to the credibility of health services (24) and a key determinant influencing health service utilization [15]. Therefore, there is need for government to improve the quality and availability of services both personnel, diagnostic equipment and frequent supply of medicines and other health services in order to improve access to services as well as wellbeing of the population which will enable the people to make better choices for health care.

### Sub- analysis for those who sought care after the first provider

At sub-analysis for those who sought care after the first provider, we found that females were more likely to seek health care at a government facility after the first provider than from a private facility. These findings may be attributed to differences in health seeking behaviour between females and males and/or economic independence between the genders in these communities that influence their choice of provider. A study in Uganda [20] found similar results that being female was associated with higher probability of using public health facilities. Contrary to our findings, a study done among tuberculosis patients in rural Ethiopia [33] found that women were less likely to visit a medical health provider than men. Therefore, sensitization on the importance of seeking health care especially targeting both sexes and empowering females economically is crucial in increasing awareness for better health care seeking practices in the management of brucellosis.

Respondents who attained tertiary education were less likely to seek health care at a government health facility after the first provider. This could be as a result of high socio-economic status of those who are more educated and are often associated with low use of public facilities which often do not have readily available services. A study by Robertson and Burge [34] found that less highly educated patients are more inclined towards choice of hospital with free services when accessing for health care. There is need to equip diagnostic facilities, increase availability and supply of medicines in government health facilities in order to improve access to care and the wellbeing of the patients or users.

## Limitations

We used a cross-sectional design and therefore no assertions can be made about causal pathways. The information collected was based on household heads'or spouses' report which is likely to lead to recall bias in case he or she forgets information and mentions what they think you expect them to say. Recall bias was minimized by interviewing only household heads or their spouses who had a family member who had suffered from brucellosis within two months before the study commenced. Another limitation was in the sample size estimation, out of a sample of 291 respondents, we managed to interview only 245 household members who had suffered from brucellosis or had family members who had been diagnosed and treated for brucellosis two months prior to the study. This could be because human brucellosis is not endemic in the area.

## Conclusions

Our study found that females and households with six to ten household members were more likely to seek health care at a government facility whereas, respondents who had attained primary education, whom the family decided where to seek health care, distance equal or more than 10 kms, those who mentioned high costs in diagnosis and treatment of brucellosis and abscence of diagnosis and treatment were less likely to choose a government health facility. Therefore, government facilities need to be equipped to attract more patients and improvements need to be made s in the five dimensions of availability, accessibility, affordability, acceptability and adequacy of health services in order to improve the ability of patients to make better choices and enhance effective control of brucellosis.
